# Enhanced Gas Sensing Performance of ZnO/Ti_3_C_2_T_x_ MXene Nanocomposite

**DOI:** 10.3390/mi13101710

**Published:** 2022-10-11

**Authors:** Qui Thanh Hoai Ta, Deepika Thakur, Jin-Seo Noh

**Affiliations:** 1 Department of Physics, Gachon University, 1342 Seongnamdaero, Sujeong-gu, Seongnam-si 13120, Korea; 2 Department of Agriculture, Forestry and Bioresources, Seoul National University, 1 Gwanak-ro, Gwanak-gu, Seoul 08826, Korea

**Keywords:** Ti_3_C_2_T_x_ MXene, ZnO, nanocomposite, gas sensor

## Abstract

A representative of titanium carbide MXene, Ti_3_C_2_T_x_ is a promising candidate for high performance gas sensing and has attracted significant attention. However, MXene naturally has a multilayer structure with low porosity, which prevents its gas-sensing activity. Zinc oxide (ZnO) has long been utilized as a gas detector. Despite its good response to multiple gases, high operation temperature has limited its widespread use as a gas-sensing material. In this study, a room-temperature toxic gas sensor was prepared from ZnO/Ti_3_C_2_T_x_ MXene nanocomposite consisting of 2D few-layered MXene and 1D ZnO nanoparticles. A simple technique for synthesizing the nanocomposite was established. The physicochemical properties of the nanocomposite were fine-controlled with more active sites and higher porosity. The sensitivity and gas-selectivity of the sensing material were closely examined. The nanocomposite showed enhanced response and recovery behaviors to toxic gases, which outperformed pure Ti_3_C_2_T_x_ MXene and pure ZnO. This study offers a practical strategy by which to increase the gas-sensing performance of Ti_3_C_2_T_x_ MXene, and expands comprehensive understanding of the gas-sensing process of ZnO/Ti_3_C_2_T_x_ p-n heterostructure.

## 1. Introduction

MXenes, also known as two-dimensional (2D) carbides, nitrides, and carbonitrides, have gained huge attention due to their unique surface properties, morphology, and potential applications [1,2,3]. MXenes are represented by the general formula: M_n+1_X_n_T_x_ (n = 1–4), where M stands for transition metals, X stands for carbon or nitrogen, and T_x_ holds for a functional group such as fluorine, hydroxyl, and oxygen [4,5,6]. Mostly, MXenes have been researched for gas sensing due to the ease of tuning the surface termination groups. Among various MXenes, titanium carbide (Ti_3_C_2_T_x_ MXene) has been studied the most, due to its high electron density and sensitive interactions with toxic gas molecules [2,7,8].

However, while MXenes have been proven to be useful, their poor stability in humid environments and loss in surface area after hydration limit its potential applications [9,10,11]. In addition, low-porosity and high operating temperatures have caused various complications during their synthesis process [12,13]. This is why there is a need for the incorporation of other materials in combination with pure MXenes.

Some efforts have been made by scientists to sensitize Ti_3_C_2_T_x_ MXene [14,15,16,17]. In our previous study, we synthesized Si-doped TiO_2_/Ti_3_C_2_T_x_ hybridstructure using a diffusion process of Si atom at 1000 °C for enhancing NO_2_ sensing activity [18]. Zhao et al. explored PANI/Ti_3_C_2_T_x_ nanocomposite to enhance ethanol sensing properties [19]. In 2021, Yang et al. studied crumpled ZnO/Ti_3_C_2_T_x_ sphere, using ultrasonic spray pyrolysis technique to improve the NO_2_ sensing performance [20]. In fact, in these previous works, a certain unilateral parameter of the sensor was enhanced. The comprehensive sensing activity of the response and recovery values should therefore be further improved [21,22].

In this work, we have highlighted the enhanced gas sensing performance of ZnO/Ti_3_C_2_T_x_ MXene nanocomposite at room-temperature. To increase the surface area of the material, we incorporated 1D ZnO nanoparticles on the surface of 2D few-layered MXene. The ZnO/Ti_3_C_2_T_x_ MXene nanocomposite significantly improved the performance for toxic gas sensing. In addition, it was found that the response and recovery time of the ZnO/Ti_3_C_2_T_x_ MXene nanocomposite outperformed the pure ZnO NPs and Ti_3_C_2_T_x_ MXene. Furthermore, the gas-sensing process of ZnO/Ti_3_C_2_T_x_ p-n heterostructure revealed better stability and performance than Ti_3_C_2_T_x_ MXene in gas sensing.

## 2. Materials and Methods

### 2.1. Materials and Chemicals

Ti_3_AlC_2_ powder was provided by 11 Technology Company (China). Zinc acetate dihydrate (Zn(CH_3_COO)_2_•2H_2_O), potassium hydroxide (KOH), and methanol (CH_3_OH) were obtained from Sigma-Aldrich (St. Louis, MO, USA). Hydrofluoric acid (HF, 48–51%) was bought from Fisher Scientific (Ward Hill, MA, USA). Ethyl alcohol (C_2_H_5_OH) was purchased from Daejung Chem (Gyeonggi-do, Korea). All the chemicals were used as provided without any further treatment.

### 2.2. Preparation of Ti_3_C_2_T_x_ MXene and ZnO/Ti_3_C_2_T_x_ Nanocomposites

First, multilayered Ti_3_C_2_T_x_ MXenes were prepared by selectively etching Al layers from the pristine Ti_3_AlC_2_ powder. Typically, a 2.0 g of pristine Ti_3_AlC_2_ MAX phase was gradually immersed into 56 mL of a concentrated HF under continuous stirring at 300 rpm in an ice bath to avoid heat generation, and the mixed solution was transferred to an oil bath at 50 °C [23,24]. After one day of reaction, the MXene multilayer structures were collected and washed several times with DI water. Subsequently, a sample of few-layered Ti_3_C_2_T_x_ MXene was prepared by ultrasonic probe sonicator (Sonics & Materials INC, Newtown, CT, USA) for around 30 min using multilayered MXene. Then, the obtained precipitate was dried overnight using a freeze-drying system (ilShinBioBase Co. Ltd., Gyeonggi-do, Korea) for further experiments.

**Figure 1:**
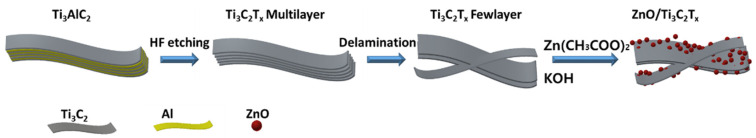
Schematic synthesis procedure of ZnO/Ti_3_C_2_T_x_ heterostructure.

ZnO/Ti_3_C_2_T_x_ nanocomposites were synthesized by a simple technique, as schematically shown in Figure 1. Briefly, varying amounts (100, 200, and 300 mg) of the few-layered Ti_3_C_2_T_x_ MXene powders were added to 25 mL of Zn^2+^ solution. A total of 340 mg of KOH was dispersed in 15 mL of methanol under heat [25]. Subsequently, the two solutions were mixed to give the final precursor solution, which was kept under heat and sonication to form ZnO nanoparticles. After 30 min of reaction, the obtained products were continuously washed to remove the byproducts and named following real ratio as ZT1 (163:100), ZT2 (163:200), and ZT3 (163:300), respectively. Moreover, the pure ZnO nanoparticles were prepared for comparison in the same parameters without any Ti_3_C_2_T_x_ MXene.

### 2.3. Characterizations and Gas Sensing Measurements

The X-ray diffraction (XRD) patterns of as-prepared samples were obtained using a high resolution X-ray diffractometer (Rigaku, SmartLab, Tokyo, Japan) equipped with 3 kW Cu Kα radiation. The morphology of all samples was collected using scanning electron microscopy (FESEM, Hitachi, S-4700, Tokyo, Japan) and high resolution transmission electron microscopy (HR-TEM, Tecnai, Hillsboro, OR, USA). The bonding states and surface characteristics of selected samples were analyzed by X-ray photoelectron spectroscopy (XPS, K_α_ plus, Thermo Fisher Scientific, Waltham, MA, USA). The Brunauer-Emmett-Teller (BET) analysis was measured using a nitrogen adsorption-desorption system (Micromeritics, ASAP 2020, Norcross, GA, USA).

To achieve gas sensing properties, concentrated suspensions of the as-prepared samples were covered on a glass substrate (10 × 10 mm). The gas sensing measurements were measured in a stainless-steel chamber (682 cm^3^) at room temperature. The real-time resistance of the samples, when exposed to the toxic target gas (R_g_) and synthetic air (R_a_), was measured through Au wires using a digital source measure unit (SMU, Keithley 2450) [26]. Five target gases (CO_2_, H_2_, CH_4_, NH_3_, and NO_2_) were used as the tested gases at high and low humidity ambient. The different gas concentrations of the toxic gas were produced by mixing it with synthetic air using a flow and pressure controller (GMC 1200). The gas sensor response value of the nanocomposite was expressed using the following equation [27]:

Response (%) = (R_g_ − R_a_) × 100/R_a_, (1)

## 3. Results and Discussion

### 3.1. Microstructures and Structural Components

**Figure 2:**
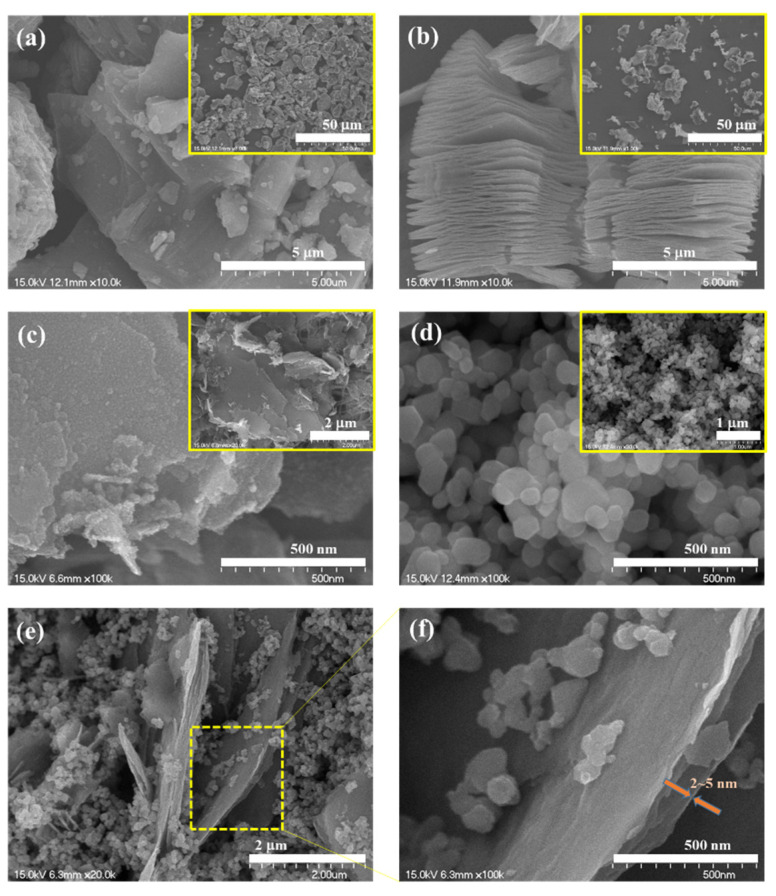
SEM images of (**a**) Ti_3_AlC_2_ MAX phase, (**b**) Ti_3_C_2_T_x_ MXene multilayer, (**c**) Ti_3_C_2_T_x_ MXene few-layer, (**d**) pure ZnO nanoparticles, and (**e**,**f**) ZnO/Ti_3_C_2_T_x_ hybrid structure. The insets show low-magnification SEM images.

Observation of morphological surfaces of the Ti_3_AlC_2_ MAX phase, Ti_3_C_2_T_x_ MXene, pure ZnO nanoparticles, and ZnO/Ti_3_C_2_T_x_ nanocomposite were analyzed using FESEM with high- and low-magnification (FESEM, Hitachi, S-4700, Tokyo, Japan). As displayed in Figure 2a, the pristine MAX phase has terraced microstructures with an average grain below 8 μm. After etching the MAX phase in concentrated HF solution, the micromorphology of multilayered Ti_3_C_2_T_x_ MXene accounts for an accordion-like sandwich, which exhibited successful Al-selective etching (Figure 2b). After sonicating the MXene multilayer for 30 min, a few-layered Ti_3_C_2_T_x_ MXene were regularly obtained. It can be observed from the inset magnified image in Figure 2c that the thickness of the Ti_3_C_2_T_x_ MXene was around 150–350 nm, which could be due to the few-layered Ti_3_C_2_T_x_ MXene. The as-synthesized ZnO displayed a particle shape with a diameter of 60–100 nm, as shown in Figure 2d. Compared to the pure ZnO nanoparticles, the few-layered Ti_3_C_2_T_x_ MXene might suppress the growth and accumulation of ZnO nanoparticles. The heterostructure could support, not only faster, but also excess diffusion paths for toxic target gases.

**Figure 3:**
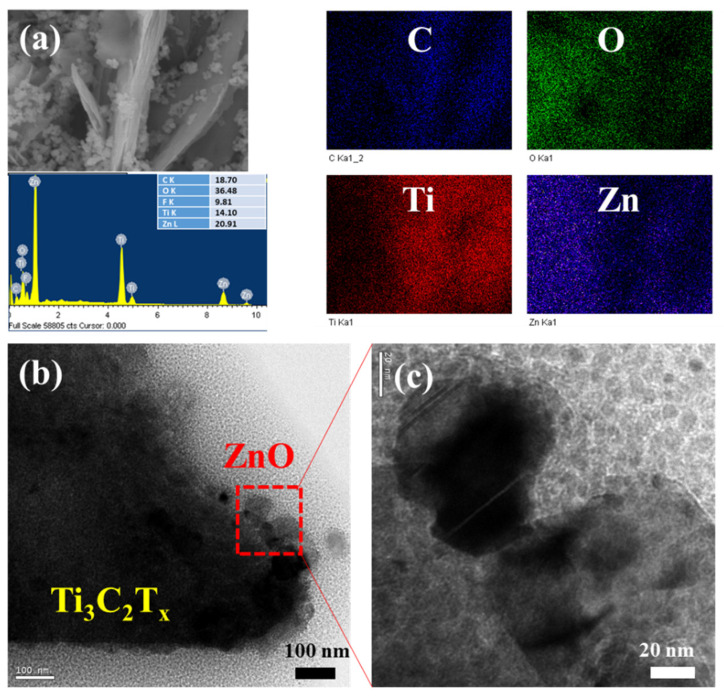
(**a**) SEM images and EDX spectrum with EDX element maps of Ti, C, O, Zn, and (**b**,**c**) HR-TEM images of ZnO/Ti_3_C_2_T_x_ heterostructure.

By focusing in further, the SEM image of ZnO/Ti_3_C_2_T_x_ nanocomposite clearly showed that the ZnO exhibited particle morphology, and featured a side anchored freely on the MXene two sides, suggesting efficient assembly between few-layered Ti_3_C_2_T_x_ MXene sheets and ZnO particles during the treatment process (Figure 2e,f). It was estimated that a single-layer Ti_3_C_2_T_x_ flake (2 nm thick) and few-layer (2–5 nm thick) flakes. The SEM and elemental mapping measurements of ZnO/Ti_3_C_2_T_x_ heterostructure (ZT2) suggested the presence of C, O, Ti, and Zn components in the nanocomposite. They displayed a highly homogeneous distribution of C, O, Ti, and Zn (Figure 3a). Moreover, the EDX spectrum also exhibited obvious C, O, Ti, and Zn elements, which were valuable for the stability and performance of the nanocomposite. As presented in Figure 3b,c, the HR-TEM images demonstrated the modification between the two interfaces, where both Ti_3_C_2_T_x_ MXenes and ZnO were cross-linked to each other. This local communication could enhance the interaction between electrons and gas molecules.

**Figure 4:**
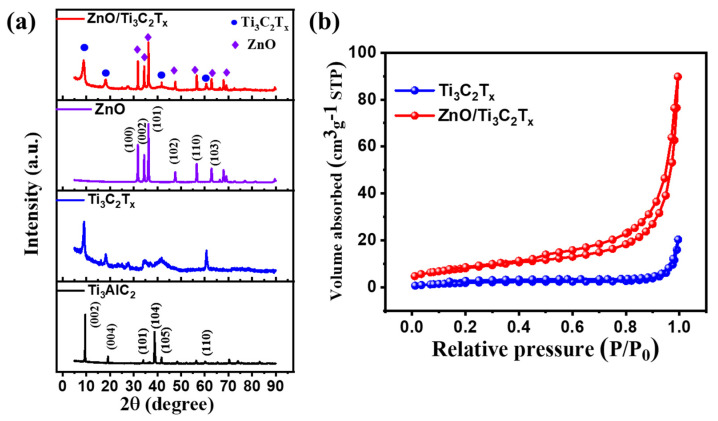
(**a**) XRD patterns of the Ti_3_AlC_2_ MAX phase, Ti_3_C_2_T_x_ MXene, pure ZnO, and ZnO/Ti_3_C_2_T_x_ nanocomposite. (**b**) N_2_ adsorption-desorption isotherms for Ti_3_C_2_T_x_ MXene, and ZnO/Ti_3_C_2_T_x_ nanocomposite.

The XRD patterns of prepared samples are displayed in Figure 4a. Well-defined diffraction peaks can be seen at 2*θ* = 9.5°, 19.1°, 36.7°, 38.7°, 38.9°, and 60.1°, which are indexed to the planes (002), (004), (101), (104), (105), (110), respectively (JCPDS card No.52-0875) [16]. After the etching development, a remarkable peak (002) in pure Ti_3_C_2_T_x_ MXene exhibits that Al was successfully corroded from the Ti_3_AlC_2_ MAX phase. The chemical bonding between each layer was decreased by increasing the *c* parameter. Meanwhile, ZnO showed preferred growth in the (101) orientation in the nanocomposite, and prominent ZnO peaks were also observed at 2*θ* = 31.7° and 34.4°, which were indexed to (100) and (002) planes, respectively. These peaks corresponded with the pure ZnO and the wurtzite structure (JCPDS card No.36-1451) [28]. The absence of any TiO_2_ peaks revealed that oxidation seemed to not occur in the combination procedure. Thus, the XRD results of synthesized samples were completely expected. As displayed in Figure 4b, both samples illustrated a type IV isotherm plot with a relative pressure from 0.42 to 1.0, indicating H3-type hysteresis circles [29]. Moreover, the BET-specific surface area (SSA) of ZnO/Ti_3_C_2_T_x_ was calculated to be 29.7 m^2^/g, which is a 3.9-fold increase compared with the SSA of the Ti_3_C_2_T_x_ MXene (7.5 m^2^/g). The results evidenced that the SSA of ZnO/Ti_3_C_2_T_x_ nanocomposite was significantly improved owing to ultra-sonication and decoration, which were expected to improve the active sites and enhance the transportation of electrons.

### 3.2. Chemical Binding States

**Figure 5:**
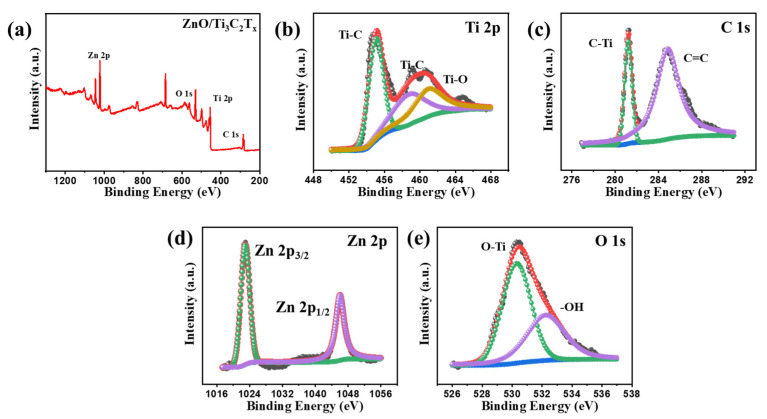
(**a**) Overall XPS spectrum of ZnO/Ti_3_C_2_T_x_ nanocomposite (ZT2). Focused XPS spectra of (**b**) Ti 2p, (**c**) C 1s, (**d**) Zn 2p, and (**e**) O 1s, respectively.

The surface chemical and binding energy of ZnO/Ti_3_C_2_T_x_ nanocomposite was observed in detail using XPS measurement for Ti 2p, C 1s, Zn 2p, and O 1s. The results are displayed in Figure 5. The overall XPS survey spectrum of ZnO/Ti_3_C_2_T_x_ heterostructure is illustrated in Figure 5a, indicating the existence of Zn 2p, O 1s, Ti 2p, and C 1s. Meanwhile, Figure 5b–e reveals the various focused XPS spectra of individual elements. Figure 5b, showing a Ti 2p XPS spectra, indicates peaks at 454.9, 458.8, and 461.08 eV, corresponding to Ti-C, Ti-C and Ti-O, which may originate from C-Ti^3+^-T_x_ and C-Ti^2+^-T_x_ with T_x_ = -OH, -O-, -F, respectively [30]. Figure 5c reveals the C 1s high-resolution spectrum, fitted using two main components at 284.7 and 281.2 eV, which could originate from graphitic C=C, and C-Ti with a surface termination group, respectively [31]. Additionally, two main electronic states of Zn 2p, shown in Figure 5d, spotted at 1045.9 and 1023.0 eV by a spin separation of 22.9 eV, are assigned to Zn 2p_1/2_ and Zn 2p_3/2_, respectively [20]. As shown in Figure 5e, the O 1s high-resolution spectrum of the nanocomposite exhibited that the two prominent binding energy peaks at 530.2 and 532.2 eV could be assigned to O-Ti and C-Ti-T_x_ (T_x_ = -OH), respectively [32]. Therefore, the above results could deduce that the optimized nanocomposite exhibited an enhanced gas sensing performance.

### 3.3. Gas Sensing Performance and Sensing

**Figure 6:**
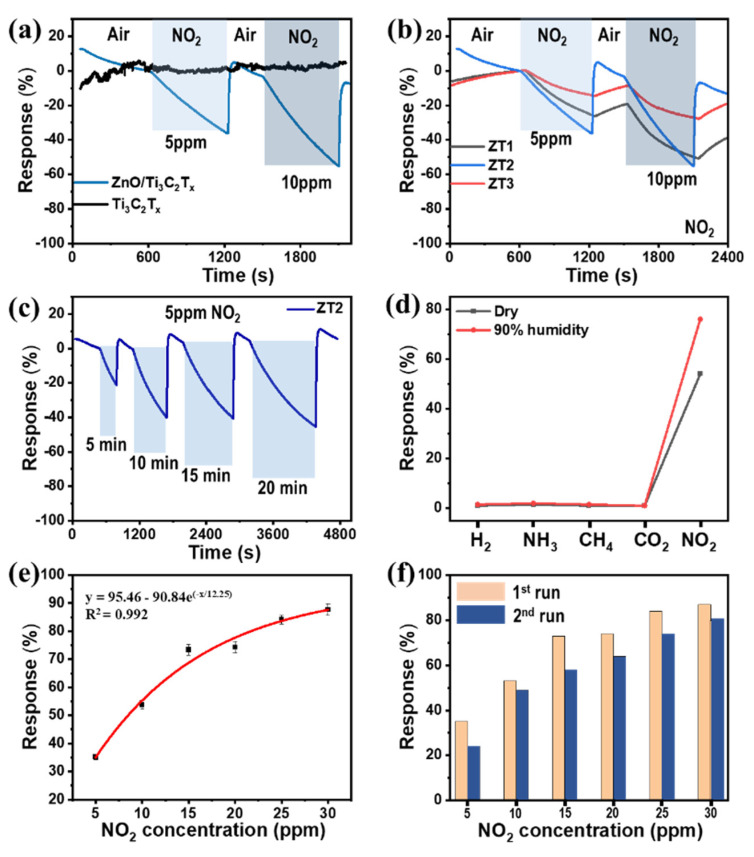
(**a**) NO_2_ response curves of pure Ti_3_C_2_T_x_ MXene and ZnO/Ti_3_C_2_T_x_ nanocomposite (ZT2). (**b**) Comparison of NO_2_ responses of ZT1, ZT2, and ZT3. (**c**) Cyclic responses of NO_2_ gas at different times. (**d**) Comparison of responses of ZT2 sample to various gases at 10 ppm concentration. (**e**) Calibration curve of sensor response versus NO_2_ concentration using logarithmic plot. (**f**) Cyclic responses of ZT2 to the different concentration of NO_2_.

In order to find the performance of the excellent gas-sensitive and selective activity of ZnO/Ti_3_C_2_T_x_ nanocomposite, the gas sensing activities of pure MXene, pure ZnO, and ZnO/Ti_3_C_2_T_x_ with different MXene weight ratios were also recorded and shown in Figure 6. Figure 6a,b illustrates the response of ZT1, ZT2, ZT3, and pure MXene sensors to NO_2_ concentrations at 5 ppm and 10 ppm, respectively. The response signal of pure ZnO did not detect at room temperature, indicating semiconductor characteristics. The response signals of all the sensors were enhanced with the increase of NO_2_ concentration, while the response of pure Ti_3_C_2_T_x_ MXene was not well developed with noise signals, due to metallic behavior. The responses to 5 and 10 ppm NO_2_ were calculated at 35% and 54%, respectively. The NO_2_ response and recovery properties of ZT2 at room temperature were more substantial than those of ZT1 and ZT3, which were inadequate (Figure 6b).

In addition, the saturation time of the ZnO/Ti_3_C_2_T_x_ heterostructure to NO_2_ was recorded. For this evaluation, the ZT2 sensor was exposed to NO_2_ at a time interval of 5 min. After 10 min, the sensing signal seemed to be saturated, as shown in Figure 6c. For further clarification, Figure 6d displays the sensing response to 10ppm of various gases (e.g., H_2_, NH_3_, CH_4_, NO_2_, CO_2_) based on the ZnO/Ti_3_C_2_T_x_ sensor. It can be observed that the ZT2 nanocomposite had the significant response to NO_2_ in comparison with other gases at low and high humidity.

In order to further explore the sensing activities of ZnO/Ti_3_C_2_T_x_ heterostructure to NO_2_ gas molecules, the ZT2 sensor was exposed to various NO_2_ concentrations ranging from 5 to 30 ppm. As illustrated in Figure 6e, the result increased with the rising concentration of NO_2_, exhibiting that the proposed ZT2 to NO_2_ molecule was logarithmic with the linear regression (R^2^) of 0.992 was acquired. Moreover, the repeatable cycle to 5–30 ppm NO_2_ molecules of the ZT2 sensor, provided in Figure 6f, reveals that the sensor possesses a repeatable characteristic. The gas sensing performance of Ti_3_C_2_T_x_-based sensors is summarized in Table 1. Although our ZnO/Ti_3_C_2_T_x_ sensor did not show a remarkable NO_2_ response compared with other sensors, it is a promising candidate for NO_2_ detection at room temperature.

**Figure 7:**
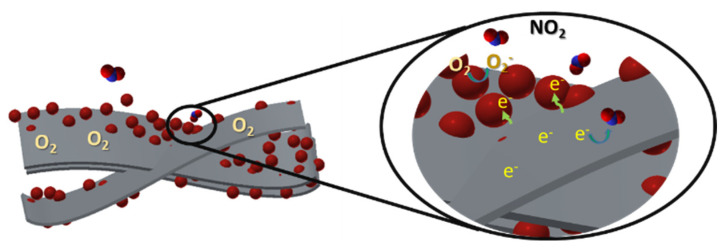
Schematic NO_2_-sensing reaction mechanism of ZnO/Ti_3_C_2_T_x_ nanocomposite.

The improved NO_2_ sensing activity of the nanocomposite was attributable to the creation of a heterogeneous p-n junction. When the ZnO/Ti_3_C_2_T_x_ MXene nanocomposite sensor was exposed to the air, oxygen molecules were covered and captured by the electrons on the material layers, as shown in Figure 7. At room temperature, most adsorbed oxygen molecules tend to create negative oxygen species (O_2_^−^, O^2−^, and O^−^) at defects and active sites of the sensor layers, which play a predominant part in improved gas sensing [33,34,35].

During the checking of NO_2_ sensing properties, it was expected that the NO_2_ gas would react with O_2_^−^ species to form NO_3_^−^ species, owing to the oxygen having lower electronegativity than the target gas [20,36,37]. Simultaneously, NO_2_ gas collected electrons and enhanced the depletion layer, thus improving the response and recovery values. Moreover, NO_2_ gas can be absorbed on the surface of p-type Ti_3_C_2_T_x_ MXene due to its oxidizing and electrophilic properties, resulting in reduced resistance [38]. Essentially, ZnO nanoparticles with an n-doping effect were decorated with a number of negative oxygen species (O_2_^−^) on the surface and supported an important role in the absorption of NO_2_ [39]. Under ambient humidity, the hydrogen bonding between NO_2_ and H_2_O molecules further promoted the chemical adsorption of NO_2_ [40]. The electron donation reaction can be described as follows [20,41,42]:

O_2_ (g) → O_2_ (ads) (2)

O_2_ (ads) + e^−^ → O_2_^−^ (ads) (3)

O_2_^−^ (ads) + e^−^ → 2O^−^ (ads) (4)

O^−^ (ads) + e^−^ → O^2^^−^ (ads) (5)

NO_2_ (g) → NO_2_ (ads) (6)

NO_2_ (ads) + e^−^ → NO_2_^−^ (ads) (7)

2NO_2_ (ads) + O_2_^−^ (ads) + 2e^−^ → 2NO_3_^−^ (ads) (8)

## 4. Conclusions

In summary, the preparation of ZnO/Ti_3_C_2_T_x_ MXene nanocomposite by effective ultrasonication was investigated. In comparison with pure ZnO nanoparticles and Ti_3_C_2_T_x_ MXene, the optimized nanocomposite achieved sensitivity and selectivity to NO_2_ gas sensing. The response to 5 ppm NO_2_ was calculated at 35% with a speedy recovery and long-term stability at room temperature. The high sensitivity NO_2_ sensing activity of ZnO/Ti_3_C_2_T_x_ MXene nanocomposite was ascribed to the active sites and defects, which were the p-n heterointerface contacts between ZnO nanoparticles and Ti_3_C_2_T_x_ MXene. The results of this combination structure pave the way for the chemical sensing of Ti_3_C_2_T_x_ MXene-based materials, thus providing a reference by which to understand the fundamentals of Ti_3_C_2_T_x_ MXene gas sensing.

## Figures and Tables

**Table 1:** The comparison of NO_2_ sensing in other materials, including Ti_3_C_2_T_x_ MXene, was explored in previous literature.

Material	Temp. (°C)	Concentration (ppm)	Response (%)
2D MoS_2_/Ti_3_C_2_T_x_ [16]	RT	10	25
3D crumpled Ti_3_C_2_T_x_/ZnO [20]	RT	20	22.5
2D/2D/2D Ti_3_C_2_T_x_@TiO_2_@MoS_2_ [31]	RT	50	55
ZnO/Ti_3_C_2_T_x_ nanocomposite *	RT	10	54

* This work.

## Data Availability

Not applicable.
